# Biogeographic Analysis Suggests Two Types of Planktonic Prokaryote Communities in the Barents Sea

**DOI:** 10.3390/biology12101310

**Published:** 2023-10-05

**Authors:** Zorigto Namsaraev, Aleksandra Kozlova, Fedor Tuzov, Anastasia Krylova, Anna Izotova, Ivan Makarov, Andrei Bezgreshnov, Anna Melnikova, Anna Trofimova, Denis Kuzmin, Maksim Patrushev, Stepan Toshchakov

**Affiliations:** 1 Kurchatov Centre for Genome Research, National Research Centre “Kurchatov Institute”, 123182 Moscow, Russia; 2 Moscow Institute of Physics and Technology, 141701 Dolgoprudny, Russia; 3 Department of Oceanology, Faculty of Geography, Lomonosov Moscow State University, 119991 Moscow, Russia; 4 All-Russian Research Institute for Civil Defense and Emergencies, 121352 Moscow, Russia; 5 Independent Researcher, 115513 Moscow, Russia; 6 Arctic and Antarctic Research Institute, 199397 Saint Petersburg, Russia; 7 Department of Geography and Hydrometeorology, Higher School of Natural Sciences and Technologies, Northern (Arctic) Federal University, 163002 Arkhangelsk, Russia

**Keywords:** Barents Sea, Arctic Ocean, Atlantification, Kola Section, prokaryote diversity, biogeography

## Abstract

**Simple Summary:**

Of all the Arctic seas, the prokaryotic communities of the Barents Sea are the most affected by climate change and are strongly influenced by microbiota from the Atlantic Ocean. Using 16S metabarcoding, we analyzed samples of prokaryotic plankton communities in the Barents Sea and found two types of communities. The origin of these communities is discussed in terms of biogeography.

**Abstract:**

The Barents Sea is one of the most rapidly changing Arctic regions, with an unprecedented sea ice decline and increase in water temperature and salinity. We have studied the diversity of prokaryotic communities using 16S metabarcoding in the western and northeastern parts of the Barents Sea along the Kola Section and the section from Novaya Zemlya to Franz Joseph Land. The hypothesis-independent clustering method revealed the existence of two distinct types of communities. The most common prokaryotic taxa were shared between two types of communities, but their relative abundance was different. It was found that the geographic location of the sampling sites explained more than 30% of the difference between communities, while no statistically significant correlation between environmental parameters and community composition was found. The representatives of the *Psychrobacter*, *Sulfitobacter* and *Polaribacter* genera were dominant in samples from both types of communities. The first type of community was also dominated by members of *Halomonas*, *Pseudoalteromonas*, *Planococcaceae* and an unclassified representative of the *Alteromonadaceae* family. The second type of community also had a significant proportion of *Nitrincolaceae*, SAR92, SAR11 Clade I, NS9, *Cryomorphaceae* and SUP05 representatives. The origin of these communities can be explained by the influence of environmental factors or by the different origins of water masses. This research highlights the importance of studying biogeographic patterns in the Barents Sea in comparison with those in the North Atlantic and Arctic Ocean prokaryote communities.

## 1. Introduction

Over the past 20 years, a new climatic regime has been developing rapidly in the Arctic Ocean [1]. Characteristic features of this new regime are reduction of sea ice area and its thickness, increase in duration of open water periods during the summer season and, as a consequence, more intensive absorption of short-wave solar radiation, leading to strong summer heating of the surface water layer. Due to the inflow of warmer and more saline waters from the North Atlantic, the process of “Atlantification” is also developing. The essence of this process is the increasing influence of ocean heat on the surface layer and ice cover due to a significant reduction in sea ice volume in the Arctic Ocean [2,3,4,5,6]. These changes are especially noticeable in the shelf seas, where the ice cover has steadily become seasonal. Positive temperature trends are also observed, especially in the Kara and Barents Seas and around Spitsbergen [7].

In the Arctic, the most intense changes occurred in the northern part of the Barents Sea. Since the mid-2000s, there has been an increase in water temperature and salinity, which is likely associated with a general reduction in the volume of sea ice in the Arctic Ocean [8,9,10]. This has resulted in decreased ice input to the Barents Sea from the north, consistent salinization, weakened density stratification, increased vertical mixing and increased heat and salt input from depth to the sea surface. These changes resulted in a further reduction of sea ice and the implementation of positive ice-ocean feedback [11]. Due to the shallowness of the Barents Sea (average sea depth 230 m), the positive feedback effect appeared here earlier than in neighboring deeper areas. This may explain why little to no ice was observed in the central and eastern parts of the Barents Sea during the 2010s [12].

The process of “Atlantification” affects the Arctic Ocean microbial community, leading to changes in its taxonomic composition [13]. There are changes in the composition of primary producers; for example, the number of blooms of the haptophytic alga *Phaeocystis* spp. more typical of the Atlantic Ocean has increased [14]. Meanwhile, satellite observations show a 57% increase in primary production in the Arctic Ocean between 1998 and 2018, leading to increased vertical carbon transport in the ocean [15]. Changes in the ratios of different taxonomic groups of microscopic eukaryotes, archaea and bacteria, as well as changes in the distribution ranges and habitats of fish and mammals have been reported [16,17,18,19,20,21].

The current number of studies examining prokaryotic diversity in the Arctic Ocean water column utilizing metagenomic methods and 16S rRNA metabarcoding is insignificant in comparison to its extensive territory and diverse hydrological conditions [22,23,24,25,26,27,28,29,30]. Nevertheless, the results already obtained allowed to the identification of the major groups of prokaryotes in the Arctic Ocean. These include primarily bacteria belonging to the groups *Alphaproteobacteria* and *Gammaproteobacteria*, as well as *Bacteroidota*. Archaea are represented in the water column in smaller numbers and are dominated mostly by the representatives of the *Thaumarchaeota* group. The prokaryotic communities of Arctic waters have a dynamic and heterogeneous composition due to seasonal changes in insolation, the presence of sea ice and nutrient concentration [28]. These factors strongly influence the diversity of prokaryotes in different parts of the Arctic Ocean [23,24,27]. An important question that has yet to receive a definitive answer is whether planktonic prokaryote communities have biogeographic distribution. Previous studies have demonstrated that planktonic prokaryote communities from adjacent basins can exhibit similar composition [27]. However, a growing amount of research, utilizing molecular approach, shows that Arctic microbial communities have biogeographic patterns [24,31,32,33]. This raises the question of the driving factors shaping these communities and controlling their distribution in the ocean.

The Barents Sea plays an important role in the formation of microbial communities in the Arctic Ocean. According to hydrological modeling, about 10 times more water enters the Arctic Ocean through the Barents Sea and Fram Strait than through the Bering Strait, which affects the temperature regime, chemical composition and microbiota of the Arctic Ocean [34]. In the Barents Sea, there is a mixing of waters from the Atlantic and Arctic Oceans, their transformation and the formation of specific Barents Sea waters [35,36]. In this region, prokaryote diversity studies using modern genomic methods were conducted only in the western part of the sea, which is most affected by the Atlantic Ocean, while the northeastern part of the sea near the Kara Sea has not been previously studied [27,28,29,30,37]. This prevents the formation of a comprehensive picture of the prokaryotic communities in this important region of the Arctic. To address this gap, we conducted a geographically broader investigation of the diversity of prokaryote communities and hydrological characteristics, which encompassed not only the western part of the Barents Sea, but also its less studied northeastern part.

## 2. Materials and Methods

**Figure 1:**
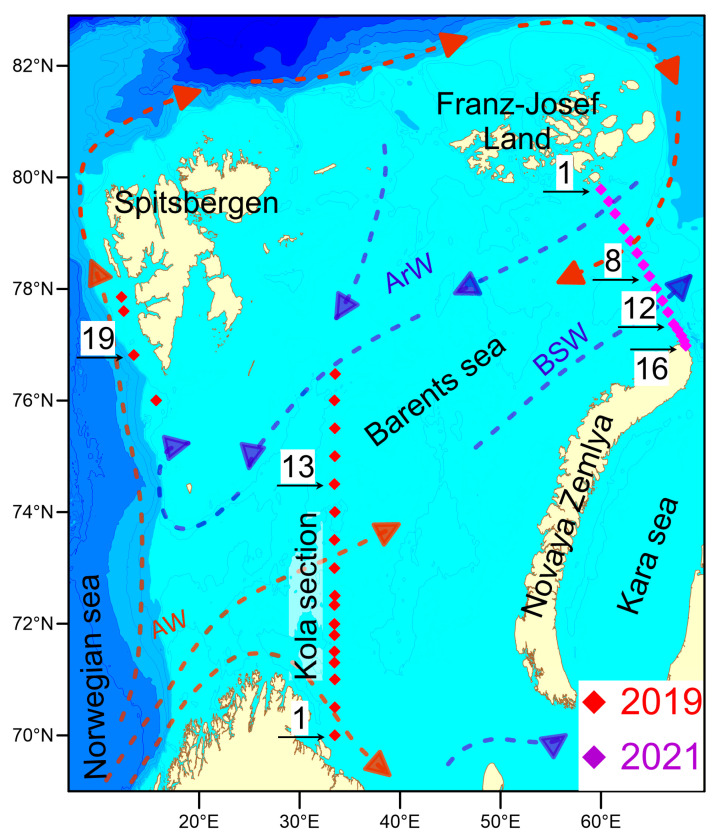
Scheme of oceanographic stations of the expeditions carried out in the summer of 2019 and 2021 in the Barents Sea. The arrows show the main ways of distribution of Atlantic waters (AWs), Barents Sea waters (BSWs) and Arctic waters (ArWs). The numbers indicate the stations at which samples were collected for microbial diversity research.

Water sampling and measurements were conducted during two cruises of the “Arctic Floating University” in 2019 and 2021. In 2019, research was conducted on the R/V “Professor Molchanov” in the western part of the Barents Sea along the section “Kola Meridian” running along the 33°30′ E meridian from 69°30′ N to 77°00′ N and being one of the world’s longest oceanic time-series [38]. Between 24 and 28 June 2019, 17 oceanological stations were performed on the “Kola Section” running along the meridian 33°30′ E from 69°59′58″ N to 76°28′20″ N, as well as at 4 stations (#18–21) located near the Spitsbergen Archipelago (Figure 1). Seawater and hydrooptical parameters were measured at all stations. Water sampling to determine microbial biodiversity was conducted at stations #1 (start coordinates 69°59′58″ N, 33°30′00″ E, 24 June 2019, 17:25 UTC) and #13 (74°30′08″ N, 33°28′48″ E, 26 June 2019, 7:45 UTC) at 10, 30 and 75 m depths. Water sampling for microbial diversity was also conducted at station #19 at 10, 30 and 75 m depths (start coordinates 76°48′51″ N, 13°29′28″ E, 29 June 2019, 10:01 UTC), located off the southwest coast of Spitsbergen.

In the summer of 2021, the R/V “Mikhail Somov” carried out a section in the northeastern part of the Barents Sea from Cape Zhelaniya (Novaya Zemlya archipelago) to Salm Island (Franz Josef Land archipelago) (Figure 1). A total of 16 oceanological stations from station #16 in the southern part of the transect (76°59′00″ N, 68°26′03″ E) to station #1 in the northern part of the section (79°47′01″ N, 59°59′05″ E) were deployed on 17–19 June 2021. At stations #1 (2 and 100 m depth, 19 June 2021, 15:00 UTC), #8 (78°13′46″ N, 64°48′27″ E, 2 and 63 m, 18 June 2019, 16:00 UTC), #12 (77°22′14″ N, 67°13′29″ E, 2 and 51 m, 18 June 2021, 3:15 UTC) and #16 (2 and 71 m, 17 June 2021, 19:20 UTC), water was sampled to determine the diversity of microorganisms. Seabird Electronics (Bellevue, WA, USA) water sampler consisting of “SBE 32 Carousel water sampler” equipped with twelve 5-L water bottles and Seabird Electronics CTD Probe “SBE 19 plusV2” for measuring temperature, pressure and electric conductivity (salinity) of seawater was used for oceanographic measurements and water sampling. Oceanological data were visualized using a software package “Ocean data view” [39]. Measurement of photosynthetically active radiation (PAR, 400–700 nm) and determination of the euphotic zone depth, at which 1% of the sea surface PAR remains were performed using Li-COR (Lincoln, NE, USA) LI-192 photometer and Data-Logger Li-1400 [40]. The airborne sensor (LI-190, LI-COR) was placed on an unshaded horizontal platform. The flux of incoming solar radiation and solar radiation penetrating to a fixed depth was recorded synchronously. Water transparency was also measured using a 30-cm diameter white Secchi disk (“UfaPribor”, Ufa, Russia) lowered to ½ the relative transparency depth. Current weather conditions (cloud cover score, sun disk condition, horizontal visibility range and sea surface condition) were recorded during the measurements. Water samples of 4 L for DNA extraction and metagenomic analysis were filtered onto sterile 47 mm Millipore (Burlington, MA, USA) GSWG047S6 filters with a pore size of 0.22 um. Filters were flash-frozen and kept at −20 °C until analysis.

DNA was extracted using Qiagen PowerLyzer PowerSoil kit (Qiagen, Hilden, Germany) according to manufacturer’s instructions. The amplicon libraries of the hypervariable V4 region of the 16S rRNA gene were prepared using the two-stage PCR strategy, as described previously [41]. Briefly, approximately 2 ng of environmental DNA and two negative controls were used for the first round of amplification with fusion primers, containing partial TruSeq adapter, heterogeneity spacer [42] and rRNA primers 515F [43] and Pro-mod-805R [44]. Amplification was performed by CFX96 Touch Real-Time PCR Detection System (Bio-Rad, Hercules, CA, USA) with the parameters described previously [41]. Amplificated mix, diluted 2–5 times (depending on Ct) was used as a matrix for the second PCR with double index-containing primers [45]. Each PCR was performed in two replicates, resulting in 34 V4 amplicon libraries. Libraries were checked with agarose gel and pooled in equimolar amounts. The final pool was cleaned with AMPure XP beads (Beckman Coulter, Brea, CA, USA), according to manufacturer’s instructions. Libraries were sequenced with the MiSeq™ Personal Sequencing System (Illumina, San Diego, CA, USA) using the 156-bp paired-end reads.

Identification of target amplicon reads in the total read pool was performed by cutadapt [46]. Read pairs not containing the primer sequence were excluded from further analysis. Demultiplexing was carried out using the deML software package [47] with parameters excluding mismatches in the index sequences. Further analysis, including read filtering (maxEE = 0.5), merging (minOverlap = 9) and chimera removal and amplicon sequence variant (ASV) reconstruction were performed according to publicly available DADA2 workflow (https://benjjneb.github.io/dada2/tutorial.html, accessed on 25 September 2023) [48]. Taxonomy was assigned with naïve Bayesian classification using Silva138 16S rRNA database [49]. ASV phylogenetic tree was constructed using msa library with clustalW [50]. After tree construction, the phyloseq object including abundance table, taxonomy table, ASV sequences and phylogenetic tree was created and used for all further analysis steps. In silico rarefaction analysis, ordination, alpha- and beta-diversity analysis was performed using microeco R package. Nonmetric multidimensional scaling (NMDS) ordination of samples was performed using a unifrac phylogeny-based distances [51]. Analysis of determining the optimal number of clusters was carried out using the package NbClust [52]. The following k-means cluster was made with native R package “stats”.

## 3. Results

### 3.1. Determination of the Seawater Parameters

**Figure 2:**
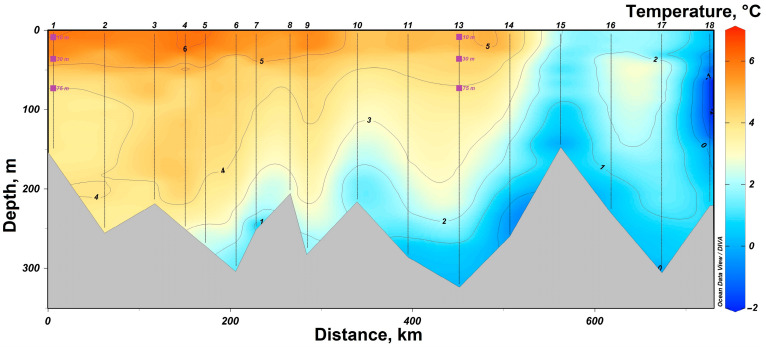
Temperature distribution in the section “Kola meridian” according to the expedition data of 2019. The symbols indicate the horizons of sampling and sampling depth at stations #1 and 13.

**Figure 3:**
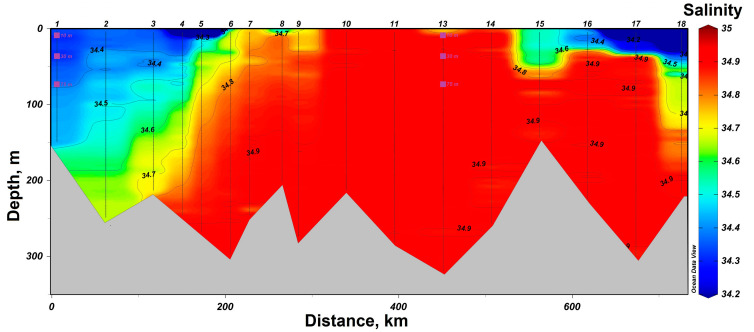
Salinity distribution in the section “Kola Meridian” according to the expedition data of 2019. The symbols indicate the horizons of sampling and sampling depth at stations #1 and 13.

In 2019, research was conducted in the western part of the Barents Sea. In the southern part of the section, station #1 was sampled in the area of the coastal branch of the Nordkapp Current, where high temperatures ranging from 4 °C at 75 m to 7 °C at 10 m were recorded (Figure 2). Waters at station #13 had lower temperature values, but its location was closer to the Atlantic water core with salinity of 34.9 at horizons deeper than 100 m. The 75 m, 30 m and 10 m horizons were characterized by lower salinity values of 34.8 (Figure 3). Thus, both investigated stations on the section are located in the areas of Atlantic water inflow to the Barents Sea basin. All sampling horizons are located above the Atlantic water core. The waters at 10-m horizon are subjected to seasonal desalinization due to ice melting, where salinity value decreases to 34.7. Nevertheless, at the northernmost station #18, located behind the polar front, it was possible to reveal arctic water intrusion at a depth of about 100 m with a temperature of less than −1 °C (Figure 2).

In addition to the Kola section, sampling was carried out at 10, 30, and 75 m depth at station #19, located off the southwestern coast of Spitsbergen (Figure 1). The West Spitsbergen Current is located in the area of the investigated station. A maximum salinity value of 34.95 was recorded at 100 m. There is also a local maximum temperature at this depth reaching 3.8 °C.

**Figure 4:**
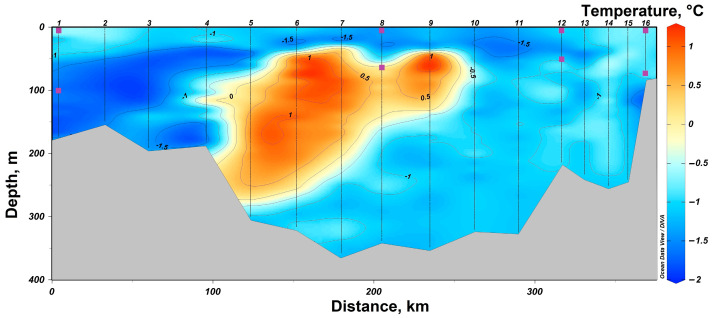
Temperature distribution in the section in the northeastern part of the Barents Sea according to expeditionary data in 2021. The symbols indicate the horizons of sampling and sampling depth at stations #1, 8, 12 and 16.

**Figure 5:**
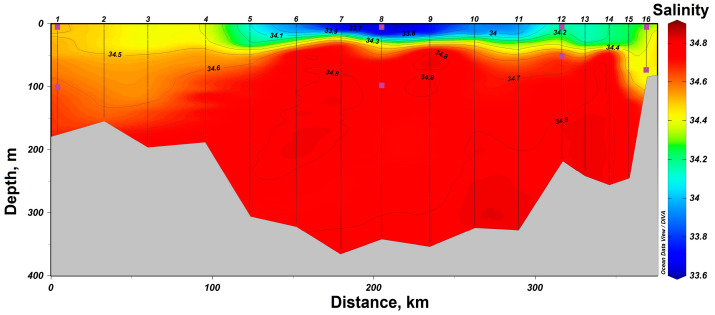
Salinity distribution in the section in the northeastern part of the Barents Sea according to the expeditionary data of 2021. The symbols indicate the horizons of sampling and sampling depth at stations #1, 8, 12 and 16.

In 2021, a section from Cape Zhelaniya (Novaya Zemlya) to Salm Island (Franz Josef Land) was performed in the northeastern part of the Barents Sea (Figure 1). In the southeastern part of the section, at stations #11–16, the Barents Sea waters are carried by the West Novaya Zemlya Current into the Kara Sea. These waters are characterized by negative temperatures not reaching the freezing point from −1.5 °C to −0.5 °C (Figure 4) and high salinity of up to 34.8. At the surface, these waters have a lower salinity (34.1–34.4) (Figure 5). In the deeper layers of the central part of the section at stations #4–10, there is an inflow of chilled Atlantic waters passing around the Spitsbergen and Franz Josef Land archipelagoes from the north. These waters are characterized by a positive temperature of up to 1.0 °C and an elevated salinity of up to 34.8. The surface layers of these waters have a lower salinity (33.7–34.2). In the shallow northwestern part of the section at stations #1–3, there is an inflow of Arctic waters with a salinity around 34.4–34.7 (Figure 1 and Figure 5). Their distinctive feature is the temperature is close to freezing point below −1.5 °C (Figure 5). At stations #5 and 13, there was a partial ice cover with a 1/10 to 2/10 ice concentration. Other stations were free of ice.

Thus, the stations selected for microbial diversity studies belong to areas occupied by different water masses. Stations #1, 13, 19, collected in 2019, are located in the area of the Atlantic waters. Station #1, collected in 2021, is located in the area of chilled Arctic water inflow. Station #8 (2021) is located in the zone of mixing of Barents Sea and chilled Atlantic waters. Stations #12 and #16 (2021) are located in the area of distribution of Barents Sea water formed as a result of winter convection in the Barents Sea.

**Figure 6:**
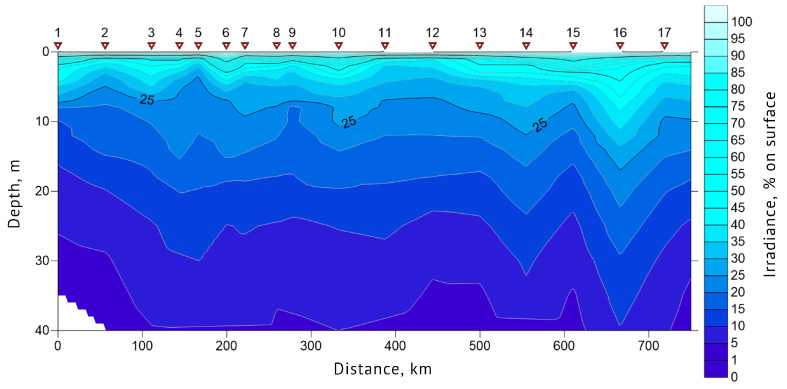
Euphotic zone light profile of the section “Kola Meridian” according to the expedition data of 2019. Red triangles indicate the location of stations #1–17.

**Figure 7:**
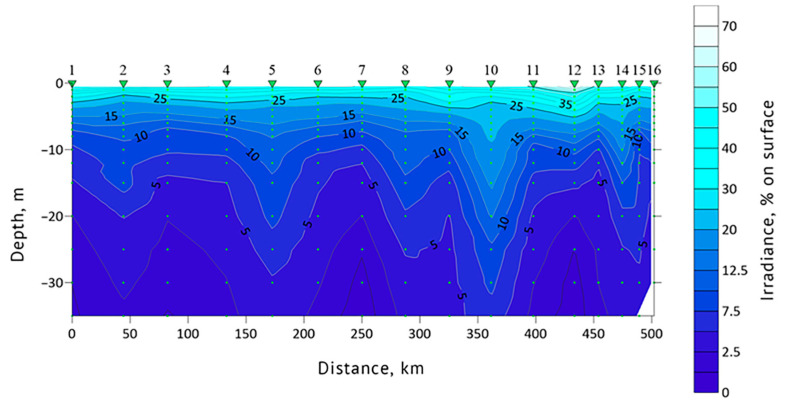
Euphotic zone light profile of the section in the northeastern part of the Barents Sea according to the expeditionary data of 2021. Green triangles indicate the location of stations #1–16.

Euphotic zone depth and water transparency (Secchi depth) data were obtained for each sampling station. In 2019, Secchi depth values at stations #1, 13 and 19 were 14, 14 and 9 m, respectively. The euphotic zone depth was 39.5 m at station #1, 47.8 m at #13 and 27 m at station #19. In 2021, Secchi depth values at stations #1, 8, 12 and 16 were 10, 15, 9 and 14 m, respectively. Euphotic zone depths at these stations were 33.4, 46.3, 27.1 and 35.4 m, respectively. The general features of the underwater light intensity in the upper layer of the sea are shown in Figure 6 and Figure 7. The highest water transparency was registered in the areas with drifting ice, such as at station #16 of 2019 and 10 of 2021.

### 3.2. Analysis of Prokaryote Diversity Using 16S Metabarcoding

The microbial communities inhabiting the water column of Barents Sea were analyzed by high-throughput sequencing of V4 hypervariable region amplicons. In total, seventeen samples of filtered water in two PCR replicates were analyzed. After primary analysis, including read filtering, merging of the overlapping read pairs and removal of chimeric sequences, approximately 340,000 reads were suitable for further analysis. The convergence of PCR replicates, checked by Bray–Curtis distances based NMDS ordination, allowed merging of replicates for further analysis steps (Supplementary Figure S1). Readcount for merged samples was in the range of 8059–44079 reads/sample. Rarefaction analysis, performed with vegan package, showed that all the samples were sequenced with sufficient depth (Supplementary Figure S2).

#### 3.2.1. Hypothesis-Independent Clusterization of Samples by Community Type

The analysis of the number of community types, presented in the dataset was performed with the NBclust package, allowing determination of the optimal number of clusters using different indexes and clustering algorithms. Using NBclust on the CLR-normalized abundance table of all ASVs, two types of microbial communities were identified in the water column of the Barents Sea. Samples were assigned to different clusters using the k-means method implemented in base R. The first cluster included samples taken in 2019 at the stations located in the western part of the Barents Sea and station #1, located in the northwestern part of the 2021 section. Samples from stations #8, 12 and 16 collected in 2021 were located closer to Novaya Zemlya and belong to the second cluster (Supplementary Figure S3).

#### 3.2.2. Beta-Diversity

**Figure 8:**
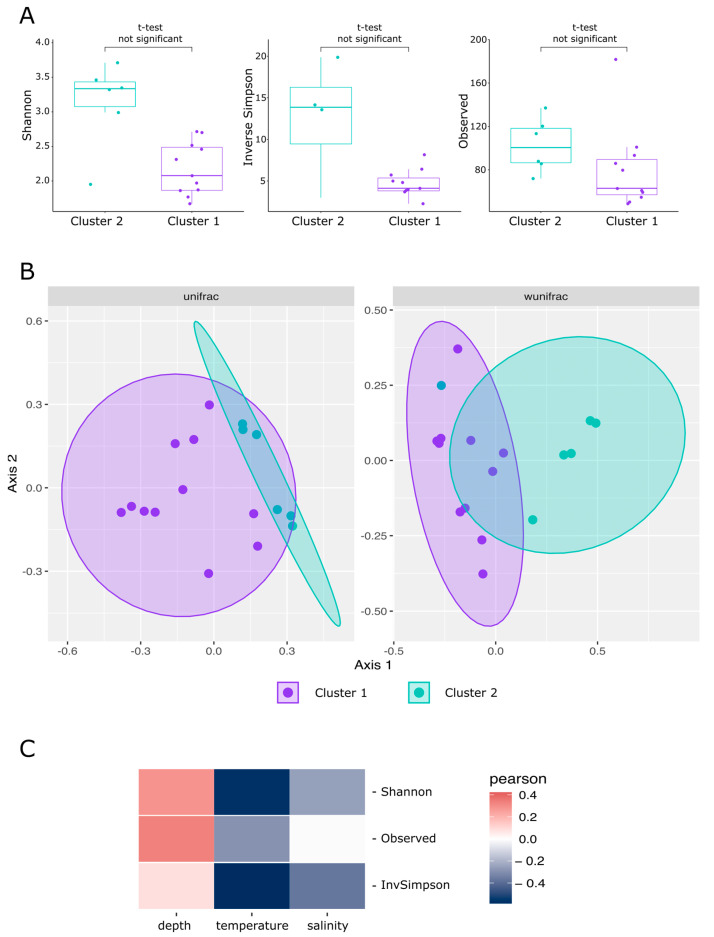
Visualization of alpha-diversity and beta-diversity analysis of prokaryote communities of the Barents Sea: (**A**) boxplots of diversity metrics compared between two clusters in Barents Sea; (**B**) principal coordinates analysis (PCoA) ordination of variation based on weighted unifrac and wunifrac distance; (**C**) heatmap of Pearson’s correlation coefficients between alpha-diversity metrics and environmental parameters.

The analysis of multivariate homogeneity of group dispersions based on Bray–Curtis distances, performed using the betadisper function of the vegan package, showed that dispersions in both clusters were homogeneous (*p*-value > 0.01). This makes it possible to apply permutational analysis of variance to test the hypothesis of differences in the composition of the microbial communities of these groups. Permutational analysis of variance, performed by adonis function, showed statistically significant differences in the microbial community composition of cluster 1 and cluster 2 samples (*p*-value < 0.01). The geographic location of sampling sites explained more than 30% of the difference in the microbiome (R2 = 0.34). NMDS ordination of samples also showed distinctive clusterization of cluster 1 and cluster 2 samples, causing the difference in the centroid of ellipses between these two groups (Figure 8).

#### 3.2.3. Alpha-Diversity

Picking of ASVs, performed with DADA2 pipeline, resulted in 617 ASVs corresponding to 185 different genera. 572 ASVs, corresponding to 174 different genera, were presented in significant amounts (more than 1% of classified reads at least in one sample). *T*-test did not show significant difference between alpha-diversity metrics in cluster 2 samples compared to cluster 1 (*p*-value > 0.01). Cluster 2 samples were characterized by higher diversity and share of specific microorganisms than cluster 1 samples. Thus, 247 cluster 2-specific ASVs, representing 32.5% of total reads were detected in cluster 2 part of the dataset, as opposed to 100 ASV, representing 1.2% of total reads, detected in cluster 1 part of the dataset (Figure 8 and Supplementary Table S1). Analysis of alpha-diversity metrics correlation with environmental parameters, including sampling depth, water temperature and salinity, showed that the diversity increased with the increase in depth, while it decreased with the increase in temperature and salinity. However, all these correlations were not statistically significant (*p*-value > 0.05).

#### 3.2.4. Prokaryote Community Composition

**Figure 9:**
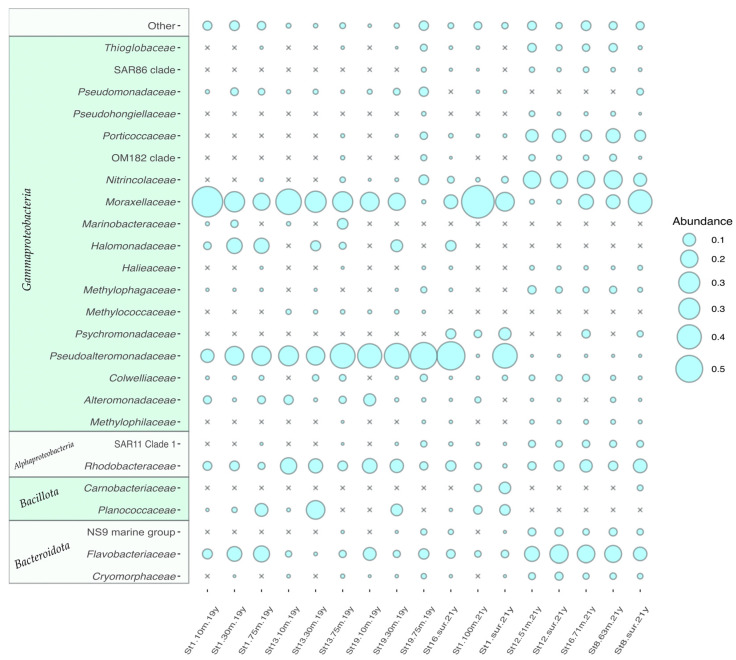
16S rRNA gene sequence-based microbial abundance analysis at the phylum level. Phyla, with less than 1% of representatives, were grouped in “Other”. The relative abundance of phyla in microbial communities is proportional to the circle area.

Bacteria dominated over Archaea in all analyzed samples. The latter was represented by ‘*Candidatus* Nitrosopumilis’, ‘*Candidatus* Nitrosopelagicus’ and unidentified Marine Group II *Thermoplasmata*, reaching maximum 0.4% of the total reads. Bacterial fraction was dominated by representatives of three phyla: *Pseudomonadota*, *Bacillota* and *Bacteroidota*. The most abundant phylum was *Pseudomonadota* (from 49.81% to 92.83% in cluster 2 samples and from 70.99 to 96.54% in cluster 1) (Figure 9). In most cases, *Pseudomonadota* were mainly presented by *Gammaproteobacteria*, however the set of gammaproteobacterial genera was different for cluster 1 and 2 samples. There were four dominating gammaproteobacterial ASVs in cluster 1 samples. The ASV1, assigned to *Pseudoalteromonas* sp. was found in most of cluster 1 samples, showing abundance in a range from 10.93% to 51.47%. ASV2 and ASV3 were associated with *Psychrobacter* sp. and found in most samples from cluster 1 (0.55–65.10%), while in cluster 2 samples *Psychrobacter* was presented by ASV7, probably corresponding to another uncultivated species. Another gammaproteobacterial ASV common for cluster 1 was classified as *Halomonas* sp. (1.9–13.04%) and unclassified representative of the family *Alteromonadaceae* (0.2–8.8%). The major clades of *Gammaproteobacteria* of cluster 2 were unclassified representatives of *Nitrincolaceae* family, namely ASV12 (up to 9.3%, 2% on overage) and ASV8 (up to 16%, 6% on average). It should be noted that ASV8 was also presented in half of the cluster 1 samples as well; however, the abundance was significantly lower (up to 0.9%, 0.1% on average). SAR92, belonging to *Porticoccaceae*, also reached significant share of the Cluster 2 community (up to 8.8%, 1.3% on average). Their abundance in Cluster 1 reached only 1.9% with 0.05% on average.

*Alphaproteobacteria*, presented in cluster 1 samples, were mainly associated with representatives of *Rhodobacteraceae* family, including *Sulfitobacter*, *Yoonia-Loctanella*, *Planctomarina* and others (Figure 9), comprising 2.49–17.2% of the community. Among them, *Sulfitobacter litoralis* was the most abundant, accounting for up to 12.7% of the community. The abundance of *Alphaproteobacteria* in cluster 1 samples was much higher than in cluster 2 samples (Figure 9). In cluster 2, in addition to *Sulfitobacter* the representatives of SAR11 Clade 1a were present in abundance up to 2.8% of the community. Moreover, the representatives of Clades II, III, IV were found in the studied samples, but their share in the community was much smaller.

The second most abundant phylum after *Pseudomonadota* was *Bacteroidota*, reaching more than 25% of the total community in several samples. In samples from cluster 2, the abundance of this phylum was higher than in cluster 1. In cluster 1 samples, it was represented mostly by the members of *Flavobacteriaceae* family, *Salinimicrobium*, *Leeuwenhoekiella* and *Gillisia*. *Bacteroidota* of cluster 2 were more diverse and belong to three families, namely *Flavobacteriaceae*, *Cryomorphaceae* and NS9 marine group. The most abundant representative of *Bacteroidota* in cluster 2 was unclassified *Polaribacter* (up to 12.8%).

*Bacillota* (synonym *Firmicutes*) ASVs were also detected in significant abundance in several samples. Thus, representatives of *Planomicrobium* sp. were found in station 1 and station 13 samples taken in 2021, at 9.87% and 22.87%, respectively. Other abundant *Firmicutes* were *Planococcus* (up to 8.13%) and *Bacillus* (up to 1.61%).

Among other taxa, *Actinobacteriota*, represented mainly by *Rhodococcus* sp. showed significant abundances (up to 1.54%) in some of the cluster 1 samples. *Verrucomicrobiota* also accounted for more than one percent of the microbial community in some samples of cluster 2.

Oxygenic phototrophic prokaryotes had lower abundance than heterotrophic bacteria and were represented by unicellular cyanobacteria of the genus *Synechococcus* (up to 0.2%) and filamentous cyanobacteria of the genera *Tychonema* (up to 0.6%), *Phormidesmis* (up to 0.2%) and *Leptolyngbya* (up to 0.4%).

## 4. Discussion

Marine polar environments are still considered among the most understudied ecosystems [53]. However, in recent years, there has been an increasing number of studies using metagenomics-based methods in the Arctic Ocean, which have revealed common patterns of prokaryotic diversity in this region. Such studies have been conducted in various parts of the Arctic Ocean and Northern Atlantic, including the Barents Sea, the deep Arctic Ocean, including the Eurasian and Canadian Basins, the Chukchi Sea, the Fram Strait, the Greenland Sea and the North Sea [23,24,26,27,54,55,56,57]. A comparison of the lists of dominant prokaryote groups shows that the most widespread dominants in these communities are representatives of the *Polaribacter*, SAR11 and SUP05 groups. Among the dominants, representatives of groups such as SAR92, *Nitrincola, Sulfitobacter* and NS9 are frequently mentioned. Interestingly, ‘*Candidatus* Nitrosopumilis’ is mentioned as a dominant species in winter studies of the Barents Sea, Nansen Basin in the deep Arctic Ocean and sea-ice-covered parts of the Greenland Sea, possibly indicating its association with colder and organic matter-depleted habitats [23,27,28].

In the Barents Sea, various studies show the dominance of representatives of the *Gammaproteobacteria*, *Alphaproteobacteria* and *Bacteroidia* groups in the prokaryotic communities. Thiele and coauthors studied the diversity of prokaryotes in the Barents Sea in different seasons and showed that in winter the water is dominated by representatives of the SAR11 clade and the community of nitrifiers, namely ‘*Candidatus* Nitrosopumilis’ (Archaea) and LS-NOB (*Nitrospinia*), indicating a possibly significant role of chemolithotrophic metabolism in the community. During spring and summer, members of the *Gammaproteobacteria* (mainly members of the SAR92 and OM60(NOR5) clades, *Nitrincolaceae*) and *Bacteroidia* (mainly *Polaribacter*, *Formosa* and members of the NS9 marine group) utilized different phytoplankton-derived carbon sources [28]. A study by Aalto and coauthors showed that in the Barents Sea, sequences classified within the orders *Flavobacteriales* (relative abundance 31–50%, including *Polaribacter* and *Ulvibacter*), *Rhodobacterales* (relative abundance 30–43%, including *Sulfitobacter*, *Amylibacter*, *Yoonia-Loctanella*) and *Pseudomonadales* (relative abundance 5–29%) were dominant [27].

The analysis of the composition of prokaryotic communities sampled in the Barents Sea during our study showed the presence of two types of communities. We found that the most common prokaryotic taxa were shared between two types of communities, but their relative abundance was different. Among the dominant bacterial taxa, the representatives of three genera (namely *Psychrobacter*, *Sulfitobacter* and *Polaribacter*) were dominant in samples from clusters 1 and 2. All three genera are widely distributed in the world ocean and include aerobic heterotrophic bacteria. *Psychrobacter* within the family *Moraxellaceae* includes strictly aerobic, cold-adapted and osmotolerant chemoheterotrophic bacteria. They are mostly isolated from cold to warm, slightly to highly saline ecosystems [58]. Earlier *Psychrobacter* strains were isolated from the Arctic Ocean, including the Barents Sea, the Bering Sea, the Chukchi Sea and Prydz Bay [59,60]. *Sulfitobacter*, which belongs to the family *Rhodobacteraceae*, is a widely distributed aerobic bacterium capable of sulfide oxidation and consumption of carboxylic acids [61,62]. *Polaribacter*, a member of the family *Flavobacteriaceae*, is considered an ecologically central species within a cross-domain ocean interactome community and is identified as one of the most connected taxa [63]. It is widely distributed in polar seas and is able to utilize a wide range of organic substrates, including carbohydrates, amino acids and organic acids [54,64]. Several members of the genus possess proteorhodopsin that enhances living in oligotrophic seawaters [65].

Samples from cluster 1 were also dominated by members of four other groups: *Halomonas, Pseudoalteromonas*, *Planococcaceae* and unclassified representative of the *Alteromonadaceae* family. Representatives of the *Halomonas* exhibit high salt tolerance over a wide range of temperatures and are able to utilize diverse organic substrates [66,67,68]. *Halomonas* is frequently found in the Arctic seas, where it can be one of the dominant phylotypes (>65%) [69]. *Pseudoalteromonas* within the family *Pseudoalteromonadaceae* is one of the most ubiquitous heterotrophic marine bacteria, widely present in the polar areas [70,71,72]. It usually comprises around 2–3% of the total bacterial communities in the surface ocean [73]. Representatives of the *Alteromonadaceae* are obligate aerobic heterotrophs with large genomes that contain several degradative genes [74]. Earlier, it was suggested that representatives of this family may be destructors of extracellular polysaccharides released by phytoplankton during blooms in the North Sea [75].

Cluster 2 also had a significant proportion of the following six groups in the community: *Nitrincolaceae*, SAR92, SAR11 Clade I, NS9, *Cryomorphaceae* and SUP05. Recently, it was shown that uncultured *Nitrincolaceae* and SAR92 clade members prevailed in the Nansen Basin samples [27], in the samples collected in the western part of the Barents Sea during summer season [28] and in the North Sea spring bloom samples [75]. It was proposed that members of these groups utilize different phytoplankton-derived carbon sources after algal blooms [28,75,76]. SAR11 Clade I group is one of the most abundant prokaryotes in the world’s oceans, which can reach up to 50% of the cells in the photic zone of the ocean and also includes one of the most abundant microorganisms in the world, the extremely oligotrophic ‘*Candidatus* Pelagibacter ubique’ [33,55,77,78,79]. NS9 members, belonging to *Flavobacteriaceae*, reached high abundance in the Greenland Sea and Southern Ocean and were noted among the dominant bacterial groups in the Barents Sea [23,30,80]. *Flavobacteriaceae* are known as degraders of phytoplankton-derived particulate organic matter and polymeric dissolved organic matter [81]. Representatives of *Cryomorphaceae* were also found in the Barents, Beaufort and Greenland Seas [16,22,30]. In the Chukchi Sea, they make up 1–5% of the population based on estimates using fluorescent in situ hybridization [54]. Based on the traits of the described species and molecular survey data, members of *Cryomorphaceae* are not responsible for degradation of complex organic matter such as polysaccharides, but can use a limited range of organic acids and amino acids [82]. The SUP05/Arctic96BD-19 clade of gammaproteobacterial sulfur oxidizers (GSOs, *Thioglobaceae*) comprises both primary producers and primary consumers of organic carbon in the oceans. In aerobic conditions, *Thioglobaceae* grow heterotrophically and use osmolytes produced by phytoplankton for growth, including methylated amines and sulfonates [83]. Isolated cultures during aerobic growth are able to oxidize sulfur and reach higher final cell densities when glucose and thiosulfate are added to the media [84]. Representatives of *Cryomorphaceae* were also found in the Barents and Greenland Seas [23,28,29]. Thus, comparison of our results with the literature data shows the presence of almost all identified Arctic dominant groups in our samples. However, many of these groups in our samples had a minor share of the community.

The most interesting result found during this study is the identification of distinct clusters of prokaryotic planktonic communities. These two types of communities separated according to the geographical location of the sampling sites. The second cluster included samples collected in the central and southern parts of the strait between Novaya Zemlya and Franz Josef Land. All other samples were grouped into the first cluster.

The strait between Novaya Zemlya and Frans Josef Land has very complicated hydrographic characteristics [85]. In the northern part of the strait, there is an inflow of water from the Arctic and the Atlantic Ocean. The freshened and cold Arctic waters (temperature below 0 °C and salinity 33–34) come from the open part of the Arctic Ocean in the form of surface currents [86]. In addition to the Arctic waters, there is also an inflow of cooled Atlantic waters (maximum temperature 1.5 °C), which flow around the archipelagoes of Spitsbergen and Franz Josef Land from the north [87]. In the southern part, there is an outflow of water from the Barents Sea.

In terms of biogeography, the formation of different microbial populations can occur through the action of two processes [88,89,90]. Firstly, it is an environmental selection driven by environmental differences between basins or water masses [68,69,70,71,72,73]. Secondly, these are historical processes that are caused by limiting the dispersal of microorganisms between basins or water masses [91,92].

The analysis of environmental parameters shows that surface waters of the central and southern parts of the strait up to a depth of 30–50 m have lower salinity than waters of the northern part of the strait. This can be explained by a local freshening as a result of seasonal ice melting. It should be noted that in two stations of the strait, an ice cover (1–2/10) was detected, although not in the places of sampling for biological studies. The analysis of the ice chart also shows that two weeks before the survey (30 May 2021), there was an ice concentration up to 9–10/10 in the center of the strait (Supplementary Figure S4). Nevertheless, the hypothesis about the separation of two clusters due to the action of environmental factors meets two objections. Firstly, we did not find a statistically significant correlation between environmental parameters, such as depth, water temperature, transparency and salinity, and community composition. Secondly, the composition of prokaryotic communities in surface water and deeper, more saline horizons did not show significant differences. It should be taken into account that the water density analysis showed the presence of stratification in the strait, and the similarity of the microbiome between the surface and deeper layer could not be explained by active water mixing (Supplementary Figure S5). Water transparency data also indicate that a seasonal bloom may have begun at station #12. Nevertheless, the compositions of communities at this station and the neighboring ones are similar to each other, which also does not explain the difference between the two clusters of prokaryotic communities.

The second hypothesis considers the emergence of divergent clusters in terms of the origin of water masses. Recent studies show that this factor plays an important role in the biogeography of plankton at both global and regional scales [32,88,92,93]. The presence of cluster 1 communities in the northern part of the strait can be explained by the inflow of water from the Atlantic Ocean, with the current passing around Spitsbergen and Franz Josef Land. A second explanation could be the high level of similarity between the prokaryotic communities of the Barents Sea and the Nansen Basin, from where Arctic water enters the Barents Sea. It has recently been shown by Aalto and coauthors that the most common microeukaryotic and prokaryotic taxa were shared across the sampling locations in the Nansen Basin and the Barents Sea [27].

In the southern part of the strait, there is an outflow of waters that are formed in the central part of the Barents Sea. Therefore, cluster 2 can be hypothetically related to these waters. According to modern concepts, the formation of the Barents Sea-type waters takes place mainly in shallow areas, including the Central Bank area. The Central Bank is an elevation in the central part of the Barents Sea, over which a dome-shaped structure is formed with water of increased density [35,94]. This structure is supported by an anticyclonic baroclinic vortex around the slope, which divides the Atlantic water flow into two branches surrounding the dome-shaped structure. During the winter, more dense and cold waters are located over the Central Bank, which can lead to the formation of a specific microbial community in this area. In spring, stable stratification is established, and dense cold water flows into the bottom horizons and is carried into the deep waters of the Arctic Ocean between Franz Josef Land and Novaya Zemlya with BSW outflow (Figure 1) [95]. These waters could have been sampled by us during the 2021 survey.

This hypothesis also raises questions. First, out of six samples belonging to cluster 2, only four samples were collected in waters with Barents Sea origin, whereas two samples, also belonging to cluster 2, were collected in the area of presumed Atlantic waters. Secondly, it is unknown to what extent the selected samples could preserve patterns of biodiversity formed in the central part of the Barents Sea. According to current velocity modeling in the Barents Sea, the current velocity between the Central Bank and the section between Novaya Zemlya and Franz Josef Land varies from 2 to 20 cm/s, depending on depth and specific location [96,97]. Assuming an average velocity of 11 cm/s and a distance between the Central Bank area and the section equal to about 750 km, we find that the waters can reach the section after about 79 days from the moment of the waters cascading near the Central Bank. The growth rates of heterotrophic bacteria vary considerably, yet the average heterotrophic bacterial community growth rate in the Arctic Ocean is quite low [98]. Kirchman and coauthors estimate it to be 0.038 ± 0.047 d^−1^, which is about a 25-day generation time [99]. Taking into account the current velocity and relatively low-growth rates, it can be assumed that hypothetically, differences in taxonomic composition may have remained during the time of water transport. However, verification of this hypothesis requires research conducted directly at the Central Bank inwinter and spring.

## 5. Conclusions

We found two types of prokaryotic plankton communities in the Barents Sea. The formation of these communities can be explained by the influence of environmental factors or by the different origins of water masses. Nevertheless, both hypotheses have their shortcomings. This highlights the significance of conducting a more comprehensive examination of biogeographic patterns at the Barents Sea scale and across a broader range of North Atlantic and Arctic seas, in order to gain a better understanding of the interconnections of polar and near-polar plankton communities with other areas and the biogeographic processes that shape them.

## Data Availability

FASTQ sequences of this metagenomic sample have been deposited in the NCBI Short Read Archive under BioProject PRJNA 939236.

## Supplementary Materials

The following supporting information can be downloaded at: https://www.mdpi.com/article/10.3390/biology12101310/s1, Figure S1: Nonmetric multidimensional scaling ordination of variation based on weighted Bray-Curtis dissimilarity for comparing technical replicates; Figure S2: Rarefaction curves of Barents Sea samples; Figure S3: PCA ordination of samples based on the Euclidean distances between data points; Figure S4: Overview ice chart of the Barents Sea 2 weeks before 2021 sampling; Figure S5: The density profile of the section between Novaya Zemlya and Franz Josef Land; Table S1: Clusterization of samples.
