# Supplementary material for: Biogeographic Analysis Suggests Two Types of Planktonic Prokaryote Communities in the Barents Sea
Source: Biology (Basel). 2023 Oct 5;12(10):1310. doi: 10.3390/biology12101310 (PMC10604488; doi:10.3390/biology12101310)
Supplement: Supplementary file 1 [file biology-12-01310-s001.zip › biology-2622582-supplementary.pdf]

**Supplementary materials**

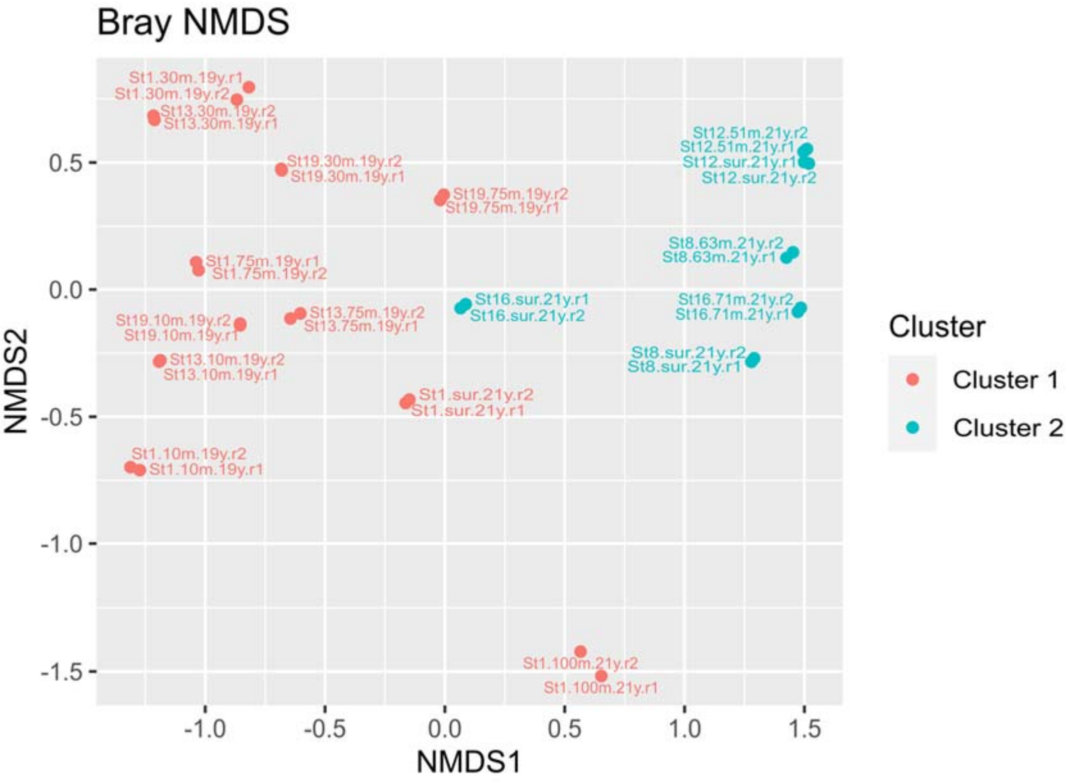


**Figure S1.** Nonmetric multidimensional scaling (NMDS) ordination of variation based on weighted Bray-Curtis dissimilarity for comparing technical replicates

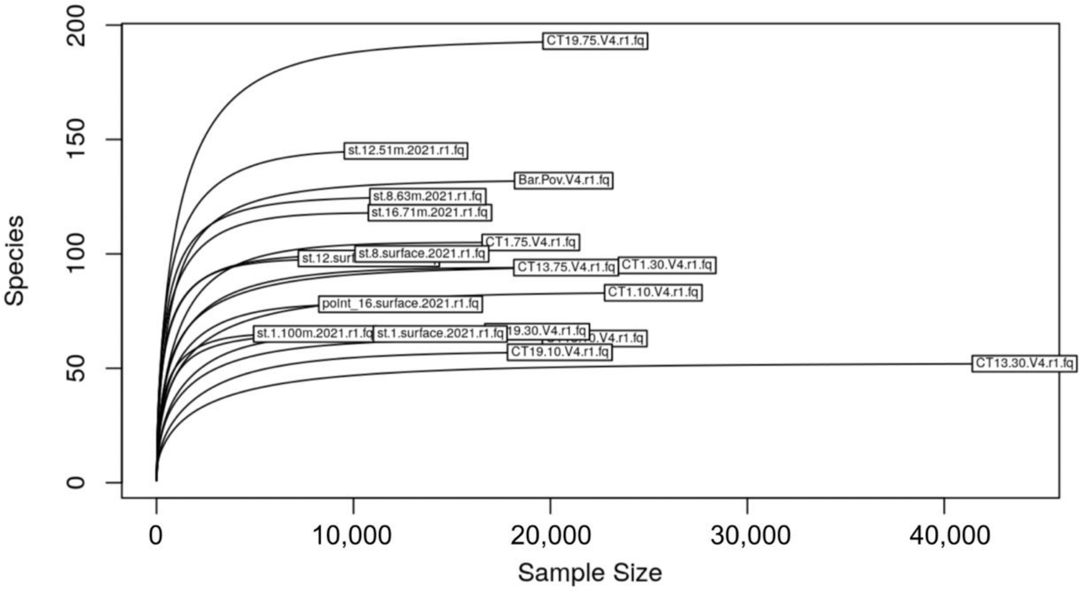


**Figure S2.** Rarefaction curves of the Barents Sea samples.

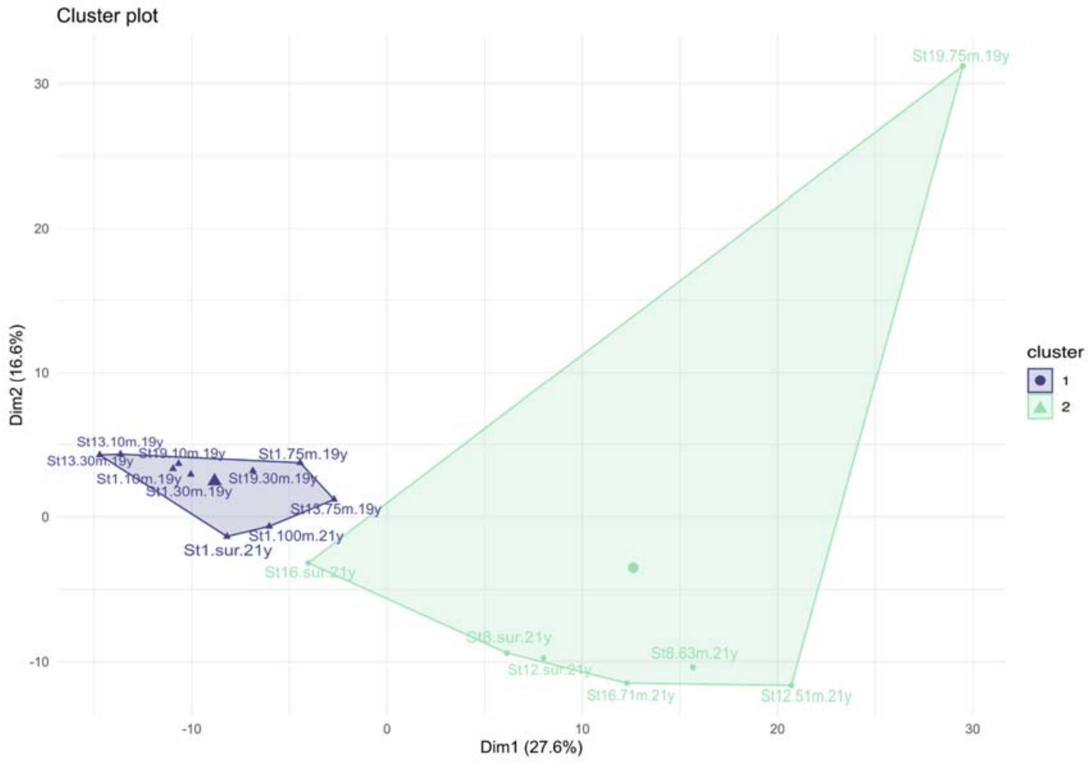


**Figure S3.** PCA ordination of samples based on the Euclidean distances between data points. Analysis showed that x-axis (Dim1, equals to PC=principal component), accounts for 27.6% of the total variation, while the y-axis (Dim2) accounts for 16.6% of the variation.

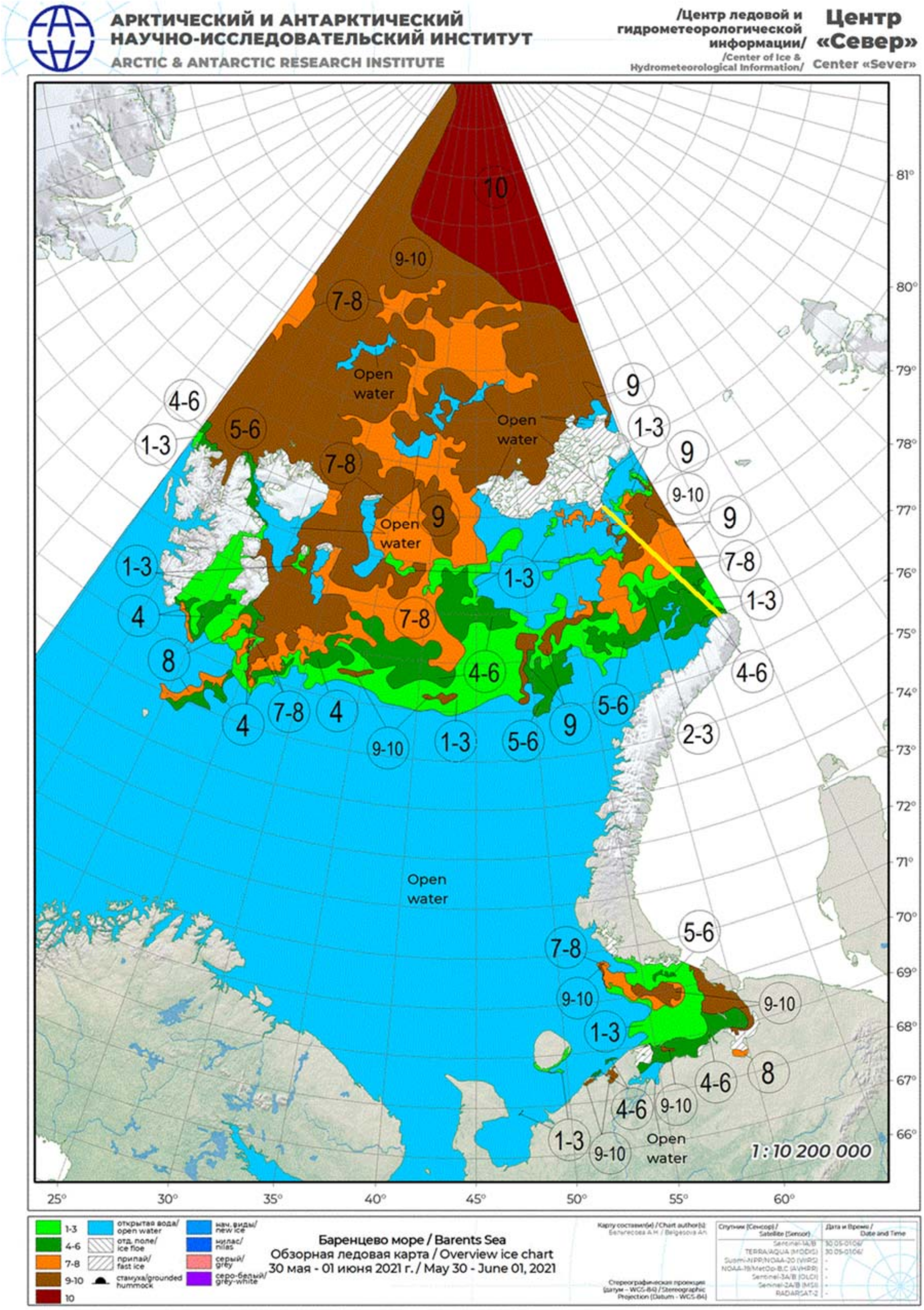


**Figure S4.** Overview ice chart of the Barents Sea 2 weeks before 2021 sampling. The yellow line shows a section between Novaya Zemlya and Franz Josef Land.

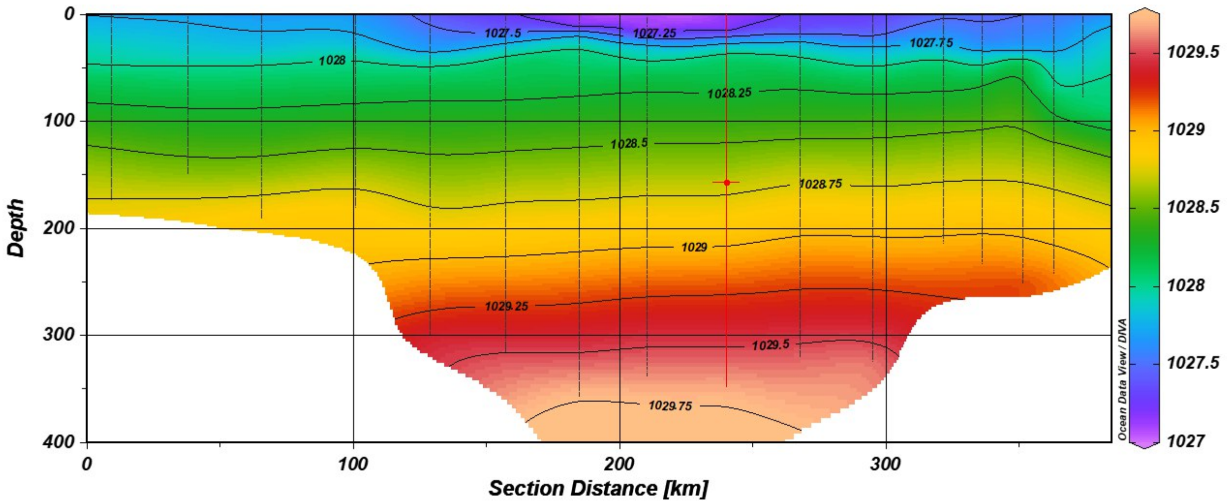


**Figure S5.**The density profile of the section between Novaya Zemlya and Franz Josef Land.

**Table S1**. Clusterization of samples

| | Shannon | Simpson | InvSimpson | Fisher | PD (Feit) | Flow |
|---|---|---|---|---|---|---|
| **St1.10m.19y** | 1.679 | 0.562 | 2.284 | 10.686 | 15.229 | Cluster 1 |
| **St1.30m.19y** | 2.525 | 0.845 | 6.440 | 12.429 | 14.875 | Cluster 1 |
| **St1.75m.19y** | 2.714 | 0.878 | 8.193 | 14.646 | 21.515 | Cluster 1 |
| **St13.10m.19y** | 1.864 | 0.740 | 3.850 | 7.936 | 12.168 | Cluster 1 |
| **St13.30m.19y** | 1.876 | 0.794 | 4.852 | 5.822 | 11.684 | Cluster 1 |
| **St13.75m.19y** | 2.133 | 0.742 | 3.871 | 12.700 | 17.082 | Cluster 1 |
| **St19.10m.19y** | 1.795 | 0.753 | 4.051 | 7.162 | 10.639 | Cluster 1 |
| **St19.30m.19y** | 2.010 | 0.763 | 4.217 | 8.544 | 13.418 | Cluster 1 |
| **St19.75m.19y** | 2.811 | 0.748 | 3.968 | 29.050 | 31.864 | Cluster 1 |
| **St1.sur.21y** | 2.367 | 0.804 | 5.105 | 8.780 | 11.713 | Cluster 1 |
| **St1.100m.21y** | 2.528 | 0.831 | 5.906 | 9.662 | 14.766 | Cluster 1 |
| **St16.sur.21y** | 1.981 | 0.669 | 3.020 | 11.115 | 19.015 | Cluster 2 |
| **St16.71m.21y** | 3.389 | 0.928 | 13.878 | 17.702 | 20.270 | Cluster 2 |
| **St12.sur.21y** | 3.241 | 0.920 | 12.572 | 14.877 | 16.873 | Cluster 2 |
| **St12.51m.21y** | 3.456 | 0.924 | 13.164 | 22.963 | 27.396 | Cluster 2 |
| **St8.sur.21y** | 3.063 | 0.880 | 8.357 | 14.652 | 16.648 | Cluster 2 |
| **St8.63m.21y** | 3.508 | 0.944 | 17.809 | 18.971 | 23.389 | Cluster 2 |
